# Comprehensive Proteomic Analysis of the Differential Expression of 83 Proteins Following Intracortical Microelectrode Implantation

**DOI:** 10.21203/rs.3.rs-4039586/v1

**Published:** 2024-03-12

**Authors:** Sydney Song, Lindsey Druschel, Niveda Kasthuri, Jaime Wang, Jacob Conard, Ernest Chan, Abhinav Acharya, Jeffrey Capadona

**Affiliations:** Case Western Reserve University; Case Western Reserve University; Case Western Reserve University; Case Western Reserve University; Case Western Reserve University; Case Western Reserve University; Case Western Reserve University; Case Western Reserve University

## Abstract

Intracortical microelectrodes (IMEs) are devices designed to be implanted into the cerebral cortex for various neuroscience and neuro-engineering applications. A critical feature of these devices is their ability to detect neural activity from individual neurons. Currently, IMEs are limited by chronic failure, largely considered to be caused by the prolonged neuroinflammatory response to the implanted devices. Over the decades, characterization of the neuroinflammatory response has grown in sophistication, with the most recent advances including advanced genomics and spatially resolved transcriptomics. While gene expression studies increase our broad understanding of the relationship between IMEs and cortical tissue, advanced proteomic techniques have not been reported. Proteomic evaluation is necessary to describe the diverse changes in protein expression specific to neuroinflammation, neurodegeneration, or tissue and cellular viability, which could lead to the development of more targeted intervention strategies designed to improve IME function. In this study, we have characterized the expression of 83 proteins within 180 μm of the IME implant site at 4-, 8-, and 16-weeks post-implantation. We identified potential targets for immunotherapies, as well as key pathways and functions that contribute to neuronal dieback around the IME implant.

## Introduction

Intracortical microelectrodes (IMEs) are devices implanted in the brain’s motor cortex that record the electrical activity of surrounding neurons ^1^. IMEs can record action potentials with single neuron resolution within an optimal range of 50–150 μm ^2^. The closer the neurons are to the implant site, the larger the magnitude of the recorded signals. In healthy individuals, “upper” motor neurons in the motor cortex send signals to “lower” motor neurons in the spinal cord, which will then innervate specific muscles in the periphery ^3^. IME recordings of upper motor neuron activity can be sent to an external system, known as a brain-computer interface (BCI), that will translate the neural recorded signals into actions that can be carried out by a device such as a prosthetic or a wheelchair. These devices have the potential to improve the lives of those with disabilities such as paralysis or limb loss ^1,4^. Commonly reported problems with IMES are their inability to maintain functionality over chronic time points and inconsistent performance between subjects ^5,6^. Chronic device failure is often attributed to the neuroinflammatory response following implantation and the subsequent neurodegeneration at the tissue electrode interface ^2^. Mitigating this inflammatory response could improve electrode performance and allow for advancements in BCI applications.

The cellular response to IME implants is well documented, as the responses of astrocytes, microglia, and neurons have been thoroughly evaluated with immunohistochemistry (IHC) ^7–9^. However, IHC is limited by the number of channels within the microscope, restricting studies to a handful of proteins per experiment. IHC is also not a fully quantitative method to evaluate protein expression, as intensity readings differ from absolute protein counts ^10^. High throughput analyses of the molecular components of the IME implant site are necessary to characterize the intricacies of the inflammatory response. Within the last 5 years, several studies have explored mRNA expression following IME implantation ^11–14^. However, there is potential for differences between mRNA expression and protein concentration ^15^. Not all mRNA molecules are translated into proteins ^16^. Additionally, a single mRNA molecule can be translated into more than one protein ^17^ meaning the mRNA to protein ratio of a specific gene is unlikely to be 1:1. Techniques used to measure the relative or absolute changes in mRNA only investigate the creation of new proteins, yet some proteins can have surprisingly long lifetimes in the body. In humans, collagen and elastin were found to have lifetimes of 117 and 78 years, respectively ^18,19^. Studies that measure mRNA expression are valuable to understand the full mechanism from transcription to translation but are not a substitute for evaluating protein expression.

This current study evaluates the expression of 83 proteins within 180 μm of the IME implant site in a mouse model, making it the first large-scale proteomic analysis of the IME neuroinflammatory response. A 180 μm radius from the implant was selected because that is approximately the length of the glial scar, so it should capture the majority of the neuroinflammation adjacent to the IME. The proteins being quantified include not just immune proteins, but also proteins from neurons and oligodendrocytes, many of which have never been evaluated in IME applications. Here, we characterized the response after 4 weeks, 8 weeks, and 16 weeks post-implantation to gain a robust understanding of the neuroinflammatory response over chronic time points.

## Results

### Overall Protein Expression:

**Figure 1:**
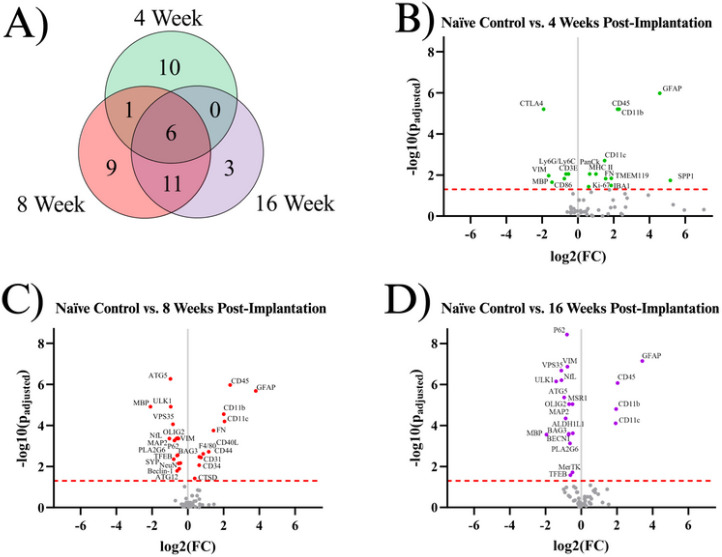
Results of the differential expression analysis. **A)** Venn diagram of the results, where the values represent the number of significantly differentially expressed proteins within the given time point. This diagram does not differentiate between up- or downregulation. **B-D)** Volcano plots of each time point, where each point is a protein in the panel. The red dashed line shows the significance threshold of p_adjusted_ = .05. All significantly differentially expressed proteins are labeled. One insignificant protein (LC3B, p_adjusted_ = .19) was omitted from plot B due to an extremely high log_2_(FC) count of 11.92.

Our starting point for investigation was to combine all of the proteins of interest investigated in this study into one complete analysis of neuroinflammatory markers. To our knowledge, our data set represents the most comprehensive assessment of neuroinflammatory protein expression following intracortical microelectrode implantation to date. Here, we first compared implanted mouse groups (4WK, 8WK, 16WK) to naïve control mice to evaluate implanted protein expression levels versus the expression levels of healthy tissue in the same region. A total of forty proteins showed differential expression within at least one temporal comparison (Fig. 1A). Table 2 shows the full list of all significant proteins, their functions, as well as a summary of when the proteins were significantly differentially expressed. There were seventeen total differentially expressed proteins in the 4WK time point. Of these seventeen proteins, ten were differentially expressed only in 4WK mice. Six proteins were upregulated in only the 4WK vs. naïve control comparison: ionized calcium binding adapter molecule 1 (IBA1), Ki-67, major histocompatibility complex class II (MHC II), pan cytokeratin (PanCk), secreted phosphoprotein 1 (SPP1), and transmembrane protein 119 (TMEM119). Four proteins were downregulated in only the 4WK vs. naïve control comparison: cluster of differentiation 3e (CD3E), cluster of differentiation 86 (CD86), cytotoxic T-lymphocyte associated protein 4 (CTLA4), and lymphocyte antigen 6 complex, locus G, lymphocyte antigen 6 complex, locus C1 (Ly6G/Ly6C) (Fig. 1A–B, Table 2). One protein, fibronectin (FN), was found to be upregulated in both the 4WK and 8WK mice but not differentially expressed at the 16WK time point (Fig. 1, Table 2).

A total of twenty-seven proteins were differentially expressed in the 8WK time point compared to naïve control mice. There were nine proteins differentially expressed only in the 8WK time point, not in the 4WK or 16WK time points. Six of those nine proteins were upregulated: cluster of differentiation 31 (CD31), cluster of differentiation 34 (CD34), cluster of differentiation 40 ligand (CD40L), cluster of differentiation 44 (CD44), cathepsin D (CTSD), and cell surface glycoprotein F4/80 (F4/80); and three proteins were downregulated: autophagy related 12 (ATG12), neuronal nuclear protein (NeuN), and synaptophysin (SYP) (Fig. 1A, C, Table 2). Seven proteins were differentially expressed at both 4WK and 8WK: cluster of differentiation 11b (CD11b), cluster of differentiation 11c (CD11c), cluster of differentiation 45 (CD45), FN, glial fibrillary acidic protein (GFAP), myelin basic protein (MBP), and vimentin (VIM), with six maintaining differential expression into the 16WK timepoint (CD11b, CD11c, CD45, GFAP, MBP, VIM). Again, FN was the only protein measured to be upregulated at both the 4WK and 8WK time points but not the 16WK time point. Of this list, only MBP and VIM demonstrated a decreased differential expression. Eleven proteins were differentially expressed in the 8WK and 16WK, but not the 4WK time point. All eleven of these proteins: autophagy related 5 (ATG5), BAG family molecular chaperone regulator 3 (BAG3), beclin-1 (BECN1), microtubule associated protein 2 (MAP2), neurofilament light (NfL), oligodendrocyte transcription factor 2 (OLIG2), sequestosome-1 (P62), phospholipase A2 group VI (PLA2G6), transcription factor EB (TFEB), unc-51-like kinase 1 (ULK1), and vacuolar protein sorting 35 (VPS35) were downregulated in the implanted animals compared to naïve control at both time points (Fig. 1, Table 2).

Three proteins were differentially expressed only in the 16WK mice: aldehyde dehydrogenase 1 family member L1 (ALDH1L1), tyrosine-protein kinase Mer (MerTK), and macrophage scavenger receptor 1 (MSR1); which were all downregulated in 16WK mice compared to naïve control.

#### Categorical Analysis of Differential Protein Expression:

Proteins that were identified as statistically significantly differentially expressed were broken down into specific categories to analyze the expression changes in specific cell types or pathways. The categories indicate proteins as being related to astrocytes or microglia, peripheral immunity, neuronal and oligodendrocyte health, and autophagy. Functions of these proteins are listed in Table 2. Many microglial and macrophage proteins are included in both the astrocyte or microglia section and the peripheral immune section because microglia and macrophages are both myeloid cells that have many overlapping markers ^20^, but macrophages are peripheral cells that infiltrate the brain tissue through damaged or leaky BBB ^21,22^. Proteins that are involved in endothelial cell function were grouped with the peripheral immune section as their downregulation could result in BBB damage and both blood-derived protein (FN for example) and blood-derived cells (macrophages for example) infiltration into the brain parenchyma.

### Indicating Proteins for Astrocytes or Microglia:

**Figure 2:**
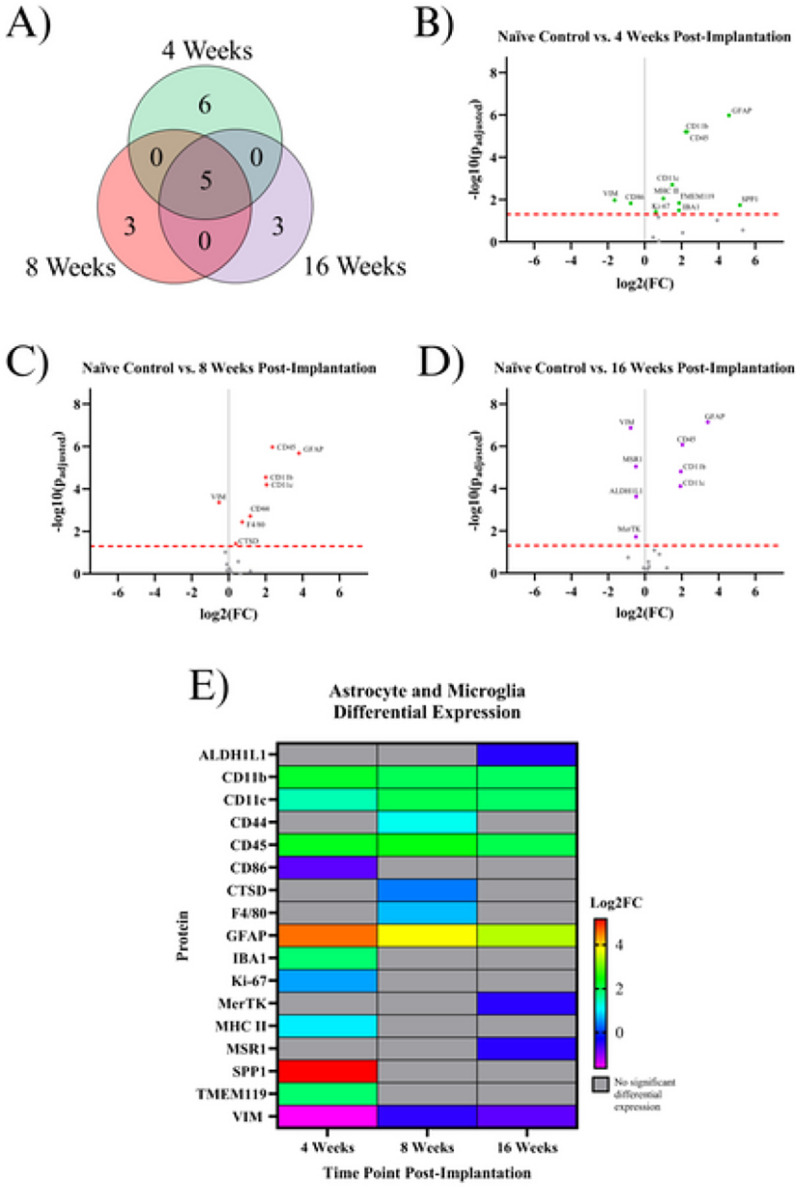
Results of the differential expression analysis of proteins associated with astrocytes and microglia. **A)** Venn diagram of the results, where the values represent the number of significantly differentially expressed proteins within the given time point. This diagram does not differentiate between up- or downregulation **B-D)** Volcano plots of each time point, where each point is a protein in the panel. The red dashed line shows the significance threshold of p_adjusted_ = .05. All significantly differentially expressed proteins are labeled. **E)** A heat map of the results, where red/yellow/green/teal represents upregulation and dark blue/purple represents downregulation compared to naïve controls. Grey indicates that there is no significant differential expression of that protein.

Astrocytes and microglia have important immune functions in the CNS, being the resident immune cells in the brain ^2^. Our analysis revealed that seventeen astrocyte or microglia-associated proteins were found to be significantly differentially expressed in at least one temporal comparison (Fig. 2). Three proteins are actrocyte-specific: ALDH1L1, GFAP, and VIM. One is microglia–specific: TMEM119. Six proteins are expressed by both microglia and macrophages: CD11b, CD11c, CD45, CD86, cell surface glycoprotein F4/80 (F4/80), IBA1. Seven proteins are expressed by astrocytes, microglia, and macrophages: CD44, Cathepsin D (CTSD), Ki-67, MSR1, SPP1, MerTK, MHC II. More information regarding the functions of these proteins can be found in Table 2.

At the 4WK time point, we saw the highest difference in expression between the implanted group and naïve control mice. Six proteins were differentially expressed in only the 4WK vs. naïve control comparison. Five of these proteins were upregulated: IBA1, Ki-67, MHC II, SPP1, and TMEM119. One protein, CD86, was downregulated. Three proteins were differentially expressed in only the 8WK vs. naïve control comparison, all upregulated: CD44, F4/80, and CTSD. Three proteins were differentially expressed in only the 16WK vs. naïve control comparison: ALDH1L1, MerTK, and MSR1, all of which were downregulated. Five proteins were differentially expressed in all three comparisons: CD11b, CD11c, CD45, GFAP, and VIM. CD11b, CD11c, CD45, and GFAP were upregulated at 4WK, 8WK, and 16WK, while VIM was downregulated at all three time points (Fig. 2, Table 2). We did not find any proteins in this sub-grouping to be differentially expressed in two, but not all three, of the examined time points.

### Indicating Proteins for the Peripheral Immune System:

**Figure 3:**
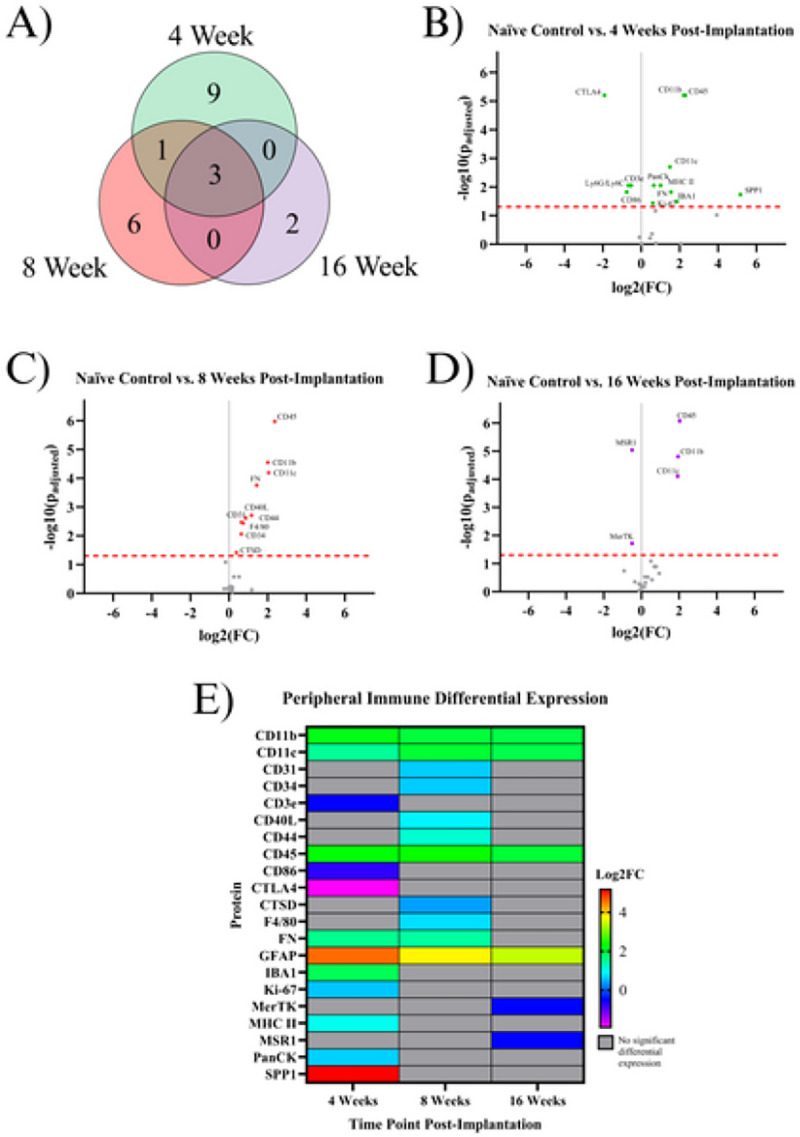
Results of the differential expression analysis of proteins associated with peripheral immunity. **A)** Venn diagram of the results, where the values represent the number of significantly differentially expressed proteins within the given time point. This diagram does not differentiate between up- or downregulation **B-D)** Volcano plots of each time point, where each point is a protein in the panel. The red dashed line shows the significance threshold of p_adjusted_ = .05. All significantly differentially expressed proteins are labeled. **E)** A heat map of the results, where red/yellow/green/teal represents upregulation and dark blue/purple represents downregulation compared to naïve controls. Grey indicates that there is no significant differential expression of that protein.

Bleeding into the injury site may cause new cell populations such as macrophages, T-cells, or neutrophils to contribute to the inflammatory response ^23^. There were 21 significant proteins associated with peripheral immunity. Five proteins are upregulated exclusively at the 4WK time point: IBA1, Ki-67, MHC II, PanCk, and SPP1. Four proteins were downregulated exclusively at the 4WK time point: CD3E, CD86, CTLA4, and Ly6G/Ly6C. One protein, FN, was upregulated at both the 4WK and 8WK time points. Six proteins were upregulated in only the 8WK vs. naïve control comparison: CD31, CD34, CD40L, CD44, CTSD, and F4/80. Two proteins were downregulated in only the 16WK time point: MerTK and MSR1. Three immune proteins were upregulated in all 3 comparisons: CD11b, CD11c, and CD45 (Fig. 3, Table 2).

### Indicating Proteins for Neurons and Oligodendrocytes:

**Figure 4:**
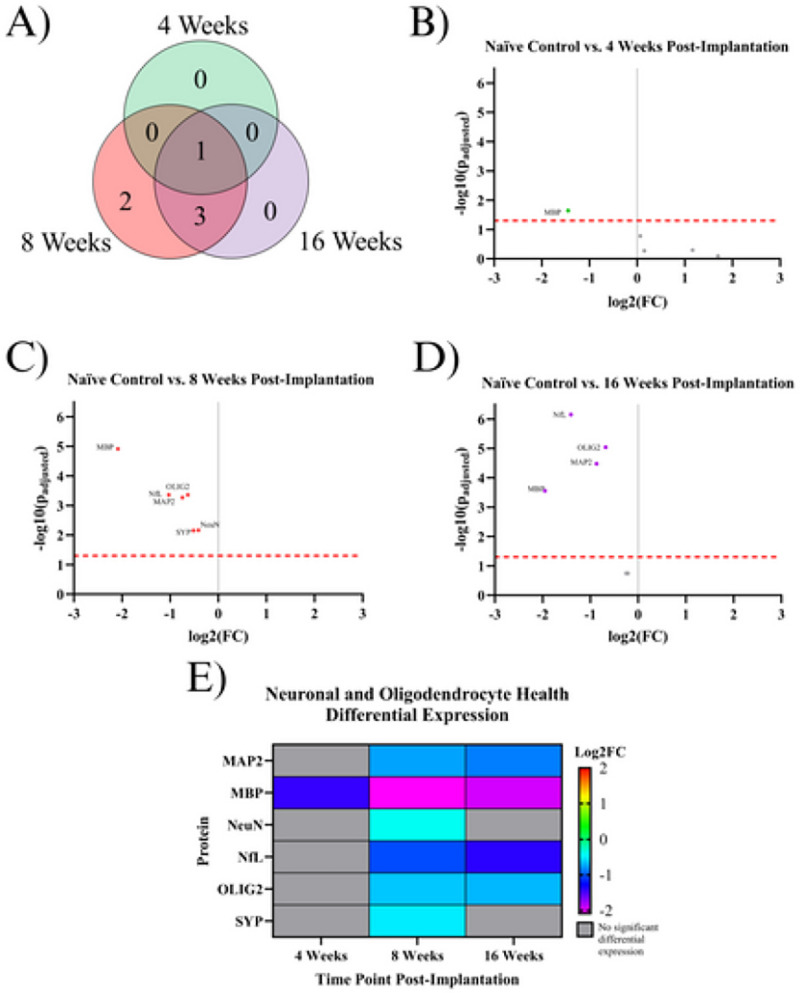
Results of the differential expression analysis of proteins associated with neurons and oligodendrocytes. **A)** Venn diagram of the results, where the values represent the number of significantly differentially expressed proteins within the given time point. This diagram does not differentiate between up- or downregulation **B-D)** Volcano plots of each time point, where each point is a protein in the panel. The red dashed line shows the significance threshold of p_adjusted_ = .05. All significantly differentially expressed proteins are labeled. **E)** A heat map of the results, where red/yellow/green represents upregulation and teal/blue/purple represents downregulation compared to naïve controls. Grey indicates that there is no significant differential expression of that protein.

Many of the proteins in the designed neural panel play a role in maintaining homeostasis in neurons, including proteins which form the neuronal cytoskeleton, synaptic vesicles, and the myelin that enhances action potential propagation ^24,25^. All proteins investigated in this study that benefit neuronal health and functionality were included in this category. Of the 83 proteins investigated in the current study, six significantly differentially expressed proteins could be classified as maintaining homeostasis in neurons or oligodendrocytes: MAP2, MBP, NeuN, NfL, OLIG2, and SYP. At the 4WK time point, MBP displayed significant downregulation in expression levels compared to naïve control. By 8WK, there is significant downregulation of all six neuronal health proteins (MAP2, MBP, NeuN, NfL, OLIG2, and SYP). Four out of the six proteins downregulated in the 8WK timepoint continued to be downregulated into the 16WK time point: MAP2, NfL, MBP, and OLIG2. Two proteins: NeuN and SYP, were downregulated at the 8WK time point but showed no differential expression at either the 4WK or 16WK time points (Fig. 4, Table 2).

### Indicating Proteins for Autophagy:

**Figure 5:**
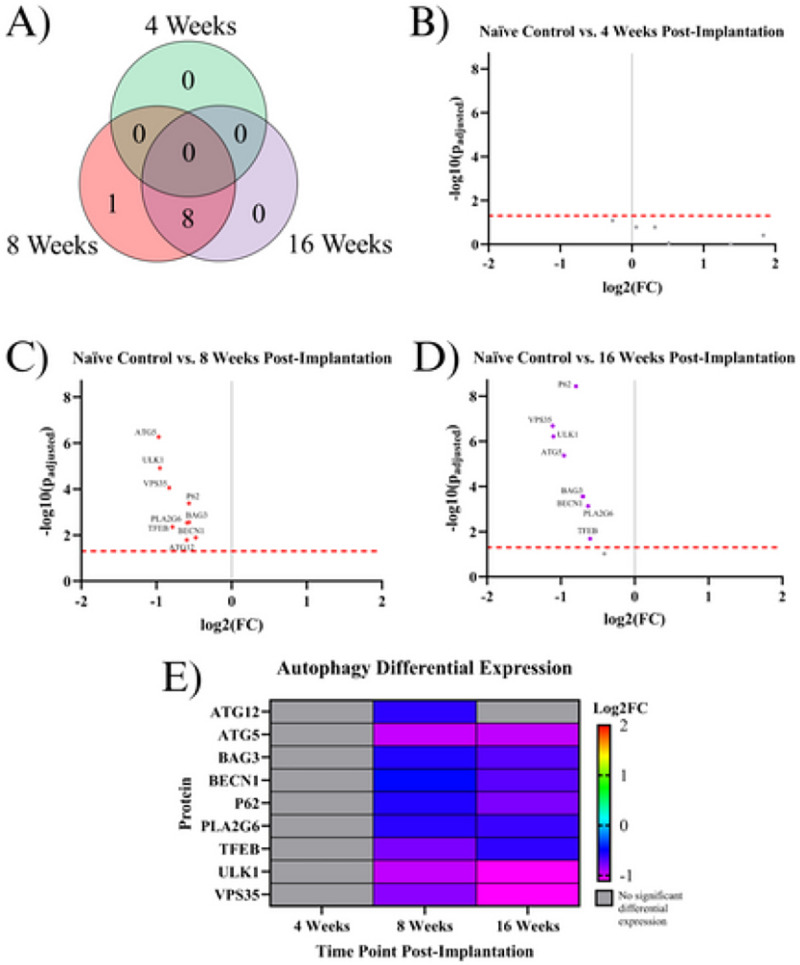
Results of the differential expression analysis of proteins associated with autophagy. **A)** Venn diagram of the results, where the values represent the number of significantly differentially expressed proteins within the given time point. This diagram does not differentiate between up- or downregulation **B-D)** Volcano plots of each time point, where each point is a protein in the panel. The red dashed line shows the significance threshold of p_adjusted_ = .05. All significantly differentially expressed proteins are labeled. **E)** A heat map of the results, where red/yellow/green represents upregulation and teal/blue/purple represents downregulation compared to naïve controls. Grey indicates that there is no significant differential expression of that protein.

Autophagy is the process in which the cell breaks down damaged and dysfunctional substances in the cytoplasm ^26^. Nine proteins associated with autophagy were found to be significantly differentially expressed in at least one temporal comparison: ATG5, ATG12, BAG3, BECN1, P62, PLA2G6, TFEB, ULK1, and VPS35. None of these proteins were significantly differentially expressed in the 4WK time point. Eight out of nine of these proteins were downregulated at both the 8WK and 16WK time points: ATG5, BAG3, BECN1, P62, PLA2G6, TFEB, ULK1, and VPS35. The one protein that was downregulated at only the 8WK was ATG12 (Fig. 5, Table 2).

## Discussion

IMEs face limitations associated with chronic failure, predominantly attributed to the prolonged neuroinflammatory response ^2^. The characterization of the neuroinflammatory response has evolved over decades, embracing advanced techniques such as genomics and spatially resolved transcriptomics ^11,12,14,27–33^. While gene expression studies enhance our broad comprehension of the interplay between IMEs and cortical tissue, there is a notable absence of reported advanced proteomic methods to explain the intricate changes in protein expression specific to neuroinflammation, neurodegeneration, or the viability of tissue and cells. This shortfall has likely hampered the development of targeted intervention strategies to enhance IME functionality.

**Figure 6:**
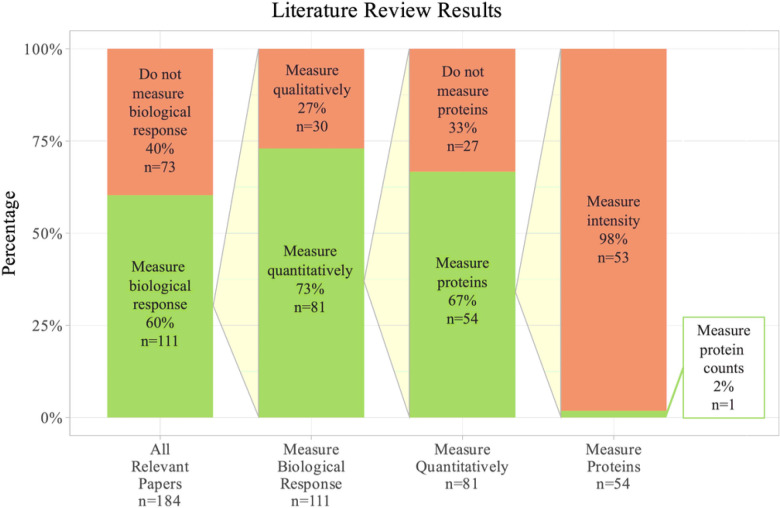
Results from literature review show of 184 papers that characterized implanted microelectrodes. Only 1 paper quantified protein expression with counts, rather than typical intensity readings. This paper measured the expression of 3 proteins. The search terms used in PubMed were: “microelectrode” AND (“biological response” OR “inflammation” OR “tissue response” OR “inflammatory response” OR “foreign body response” OR “failure”) AND (“brain” OR “cortical” OR “intracortical”).

Fig. 6 shows a review of how the biological response to neural implants is typically quantified. The search terms used in PubMed were: “microelectrode” AND (“biological response” OR “inflammation” OR “tissue response” OR “inflammatory response” OR “foreign body response” OR “failure”) AND (“brain” OR “cortical” OR “intracortical”). This search output a reasonably sized representation of the literature, but this is not an exhaustive list of all IME papers in the field. All papers from this search that were published in 2000 or later were included in the review (n = 256). The search terms were intended to target experimental papers that are developing or characterizing microelectrodes and implanting them into either the brain or live neural cells. Any papers that did not fit these requirements (n=72) were removed.

Of the remaining 184 papers, 40% (73 papers) did not mention the biological response to the implant. Of the 60% of papers (111 papers) that did mention the biological response, only 73% (81 papers) of that subgroup used any quantitative metric to determine the effect of the implant on the tissue. Of the 81 papers that used quantitative methods to characterize the biological response to the implant, 67% (54 papers) used protein expression assays to quantify the response. The 54 proteomic papers represent a relatively high number of papers using protein expression as an indication of the state of the brain tissue. However, 98% of papers (53 in total) that looked into protein expression used a method that measured intensity, such as immunohistochemistry. Analyzing intensity measurements means that the papers were not able to look at large numbers of proteins at once due to a limited number of microscope channels, and it means that the protein counts were not counting the actual protein concentration, but were instead estimating based on intensity. The average number of proteins quantified in the subgroup that used intensity measurements was 4.3 proteins. In this literature review we only found one paper that measured protein counts, and this paper quantified the expression of 3 proteins through cytometric bead arrays ^34^. Our study expands on current methods by using the actual protein counts rather than intensity readings and by quantifying 83 proteins at once.

In this investigation, we meticulously profiled the expression of approximately 80 proteins within a 180 μm radius of the IME implantation site at 4, 8, and 16 weeks post-implantation to better understand the sub-chronic and chronic neuroinflammatory response to IME implantation. Overall, of the three time points investigated in this study (4WK, 8WK, and 16WK), 4WK demonstrates the strongest changes in innate immune marker expression, while 8WK and 16WK exhibit deficits in local neurons and oligodendrocytes. (Fig. 1–5, Table 2). All ten proteins that are only differentially expressed in the 4WK vs naïve control comparison are associated with microglia, macrophages, or peripheral immune cells. The 8WK time point has the largest downregulation of neuronal health and autophagy proteins (Fig. 1C, 2, 3). By 16 weeks post-implantation (16WK), most of the 8WK effects still linger, but several proteins (ATG12, NeuN, SYP) are no longer downregulated. This could mean that the tissue is healing, but time points past 16 weeks post-implantation would be needed to confirm to what extent the tissue is able to heal.

### Proteins Associated with Astrocytes or Microglia:

The formation of the tight glial scar that encapsulates the implant is achieved through the migration and expansion of astrocytes upon activation. Two proteins involved in the cytoskeletal expansion of astrocytes are VIM and GFAP ^35^. Here, we found GFAP expression to be heavily upregulated at all time points (4WK, 8WK, 16WK), with a ~350–450% increase in implanted tissue compared to naïve control (Fig. 1). We expected a similar trend with vimentin (VIM), an intermediate filament that plays a similar role in astrocytic activation ^36^. However, we found the exact opposite to be true, as vimentin was downregulated in all three measured time points (4WK, 8WK, 16WK) (Fig. 1, Table 2). Vimentin has another role in the motor cortex: it helps to form tight junctions between endothelial cells in blood vessels of the BBB, maintaining the structure that separates the brain parenchyma from circulating blood ^37,38^. Implantation of the IME breaks these blood vessels, and the healing process is incredibly slow due to the persistence of the device in the tissue. The BBB is reported to reestablish its integrity at approximately 8 weeks post-implantation, with minor leakage continuing into 16 weeks post-implantation ^7^. With vimentin being downregulated at 4WK, 8WK, and 16WK time points, the loss around the implant site likely contributes to the leaky vasculature. It is possible that vimentin’s overall downregulation in the blood vessels is outweighing the upregulation in activated astrocytes. Aldehyde dehydrogenase 1 family member L1 (ALDH1L1) is an enzyme that regulates astrocyte metabolism, cell division, and cell growth. It was only differentially expressed (downregulated) in the 16WK mice (Fig. 1D, Table 2). The function of ALDH1L1in the CNS is not entirely known, but its downregulation in the 16WK mice may be an indication of injured or diseased state astrocytes ^39^.

In the current study, many proteins associated with microglia, specifically CD11b, CD11c, CD45, IBA1, Ki-67, MHC II, SPP1, and TMEM119, were found to be upregulated in the 4WK time point (Fig. 1B, 2, Table 2). Microglia are the main phagocytic cell type in neural tissue ^40^, and the upregulation of these proteins validates the presence of microglia at the implant site. CD11b has a known role in cell adhesion during inflammation ^40,41^. It is likely upregulated to allow for the adhesion of activated microglia and macrophages to the implanted electrodes and to one another, as they aggregate and form a thin layer on the surface of the electrode ^42^. CD11c is expressed in a subset of microglia that are believed to have neuroprotective qualities ^43^. Moreover, both CD11c and CD11b are expressed by peripheral immune cells such as dendritic cells, while peripheral macrophages also express CD11b. CD45 is considered to have low expression in microglia compared to macrophages and T-cells, and plays a role in adhesion in myeloid cells ^44^. Therefore, upregulation of CD45 could be indicative of T-cells and other peripheral immune cells at the microelectrode interface. IBA1 is involved in the membrane ruffling process and phagocytosis in activated microglia ^45^, and has been a common marker for total microglial and macrophage population in cortical tissue in immunohistochemical evaluation of the tissue-electrode interfaces ^46,47^. MHC II is a protein involved in antigen presentation that is expressed by microglia, astrocytes, and other immune cells ^48,49^. Secreted phosphoprotein 1 (SPP1), also known as osteopontin, is secreted by microglia, macrophages, and T-cells, and is involved in the toll-like receptor signaling pathway. It is pro-inflammatory and activates and recruits more microglia to the implant site ^50^. Together, MHC II and SPP1 may implicate the roles of innate and adaptive immune system in response to IME implantations. TMEM119 is a protein expressed on microglia that is mainly abundant in resting cells. Upon activation, TMEM119 concentrations are reduced in microglia ^51^. TMEM119 is upregulated at only the 4WK time point, which could indicate that by the 8WK time point the microglia have fully activated and lost the surface TMEM119 abundance. Taken together, the listed microglial and astrocytic proteins could be further investigated as a target to mitigate the inflammatory cascade. Ki-67 is a ubiquoous marker for cell proliferation, and suggest the active cell proliferation, likely by immune cells, at the site of implantation ^41,52^.

Ki-67 is upregulated in only the 4WK time point compared to naïve control (Fig. 1, Table 2). In the adult motor cortex, neurons no longer proliferate, meaning that the Ki-67 is likely expressed in astrocytes, microglia, and infiltrating peripheral immune cells, not neurons. *Ki-67*^−/−^ mice have been shown to reduce tumor growth while also inhibiting major histocompatibility complex expression (see MHC II, Table 2). However, Ki-67 is not essential for proliferation to occur ^53^. It is not known how Ki-67 targeting would affect traumatic brain injury or IME implantation, but reducing the efficiency of proliferation in immune cells as well as inhibiting major histocompatibility complexes may be beneficial to neural injuries by reducing the amplification of the inflammatory response in the first 4 weeks following the injury. Other proteins in the proliferation pathway, such as NOX4 or CSF1R, may reduce proliferation more effectively compared to *Ki-67* knockout.

#### Proteins Associated with the Peripheral Immune System

Peripheral immune protein expression can be used to quantify the extent of immune activity at the implant site. These proteins are mainly expressed by T-cells, macrophages, dendritic cells, and neutrophils that circulate through the blood and are recruited into the IME implant site. The BBB is reported to reestablish its integrity at approximately 8 weeks post-implantation, with minor leakage continuing into 16 weeks post-implantation ^7^. Peripheral immune cells can be both passively and actively recruited to the site of injury following microelectrode implantation. IBA1, Ki-67, MHC II, and SPP1 are upregulated at only the 4WK time point (Fig. 1, Table 2). All four of these proteins are found on microglia and macrophages, and were discussed in the Astrocyte or Microglia Proteins section. Four proteins are downregulated at the 4WK time point: CD3E, CD86, CTLA4, and Ly6G/Ly6C. CD86 expressed on innate cells binds and activates CTLA4, and it is believed that this binding dampens T-cell activation by keeping CD86 from binding with CD28 ^54^. The levels of CTLA4 are low in resting T-cells, and the protein is upregulated by T cells as a self-regulating mechanism of the immune system to prevent run-away inflammation ^55^. CTLA4 is downregulated, but CD40L, another protein involved in T-cell activation, is upregulated at the 8WK time point (KEGG:04660) (Fig. 1, Table 2) ^56–58^. The combined low CTLA4 and high CD40L demonstrates that at 8WK time point there might be higher infiltration of activated T-cells at the implant site.

The mice implanted with IMEs for 4WKs or 8WKs have seven proteins associated with peripheral immune cells upregulated compared to naïve control mice (Fig. 1B, C, 3, Table 2). However, by 16WK, only three of these proteins remains upregulated (Fig. 1D, 3, Table 2). The three peripheral immunity associated proteins upregulated at all three time points are CD11b, CD11c, and CD45 (Fig. 1, 3). All three of these proteins are expressed in microglia and macrophages (Table 2). Ly6G/Ly6C is expressed in both myeloid-derived suppressor cells and neutrophils ^59,60^. This data indicates the persistence of potential innate immune cells such as macrophages, neutrophils, dendritic cells until 16 weeks of this study.

The upregulation of peripheral pathways indicates the presence of specific cell types, mainly macrophages and T-cells. On the other hand, downregulation of peripheral pathways indicated that these cells do not migrate into the implant site or are selectively cleared by the 4WK time point. T-cells and other peripheral cells such as dendritic cells and neutrophils are relatively uncharacterized in the context of IME implantation, and represent a potential emerging area for immunomodulation of the neuroinflammatory response to intracortical microelectrodes. In fact, immunomodulation of many diseases and injury states though T-cell programing is becoming an emerging area for immunoengineering ^61–63^. Perhaps similar strategies can be adopted for the neural interface.

#### Proteins Associated with Neurons and Oligodendrocytes

All six measured proteins associated with maintaining the structure of neurons and oligodendrocytes: MAP2, NfL, SYP, NeuN, MBP, and OLIG2, are downregulated in at least one timepoint (Fig. 2, Table 2). The broad downregulation of markers for neuronal health indicates significant deficits in the health and functionality of both neurons and oligodendrocytes. Two neuronal health proteins: synaptophysin (SYP) and neuronal nuclear protein (NeuN), are downregulated in implanted animals at the 8WK time point and are not significantly differentially expressed at the 4WK or 16WK time points. SYP is a protein that lines the synaptic vesicles ^25^. Synaptic vesicles are used to transport neurotransmitters to the synaptic terminals. The release of the neurotransmitters from the synaptic vesicles allows for signal transmission from neuron to neuron ^64^. Decreased synapse could indicated a decrease in neurons or a decrease in synaptic connections between neurons. NeuN is a protein found in neuronal nuclei involved in mRNA splicing ^65^ and is the most common marker in the IME histology literature for neuronal health and survival ^65^. The fact that SYP and NeuN are both downregulated at 8WK and are not significantly differentially expressed at the 16WK time point could indicate neuronal healing of some capacity between 8 weeks and 16 weeks post-implantation. Similar trend of fluctuations in NeuN density in histological evaluation of the IME-tissue interface were reported by Potter *et al*. ^66^.

MAP2, NfL, MBP, and OLIG2 are all downregulated at both the 8WK and 16WK time points (Fig. 1, 2, Table 2). Neurofilament light (NfL) and microtubule-associated protein 2 (MAP2) are both proteins that make up the neuronal cytoskeleton. Degradation of the neuronal cytoskeleton has been linked to the transition between reversible and irreversible damage to brain tissue ^67,68^. NfL is downregulated by ~50% (FC= 2^−1^) in implanted animals by 8WK and ~62% (FC= 2^−1.4^) by 16WK (Fig. 1C, D). The increasing decline in NfL expression could be directly linked to decreases in neuron recording performance with time. However, NfL does not represent a target for immunomodulation approaches to mitigate IME performance. The integrity of the oligodendrocytes, which make up the myelin that allows for efficient transmission of action potentials, are also compromised following IME implantation. Myelin basic protein (MBP) is the second most abundant protein in the myelin cells of the central nervous system ^69^, and is considered to be essential for the formation of tight myelin sheaths around an axon ^70^. MBP is approximately four times more abundant in naïve control animals compared to implanted mice at 4WK, 8WK, and 16WK time points (Fig. 1B, C, D). This means that ~75% (FC = 2^−2^) of MBP is lost following IME implantation, which would significantly impact the functionality of the cortical neurons and by extension, the ability of IMES to detect single-unit activity. MBP is the only neuronal health protein that begins downregulation as early as the 4WK time point. Oligodendrocyte transcription factor 2 (OLIG2), is a protein that regulates the transcription for myelin-associated proteins. In traumatic brain injury, OLIG2 is upregulated immediately after injury and remains upregulated for up to 3 months, aiding in the remyelination of the tissue ^71,72^. In the case of IME implantation, we see the opposite effect with OLIG2 downregulation at both 8 and 16 weeks post-implantation (Fig. 1, 2, Table 2). The permanent presence of the electrode may be preventing successful remyelination.

Overall, the neuronal health proteomic data indicates that the health and functionality of neurons and oligodendrocytes is likely the lowest at approximately the 8WK time point. Degradation of the neuronal cytoskeleton (NfL, MAP2) as well as the oligodendrocytes (MBP, OLIG2) begins at the 8WK time point and continues into the 16WK mice. Some components, including SYP and NeuN, are at least partially regenerated by 16 weeks post implantation, but other components of the cytoskeleton have endured what may be irreversible damage. These structural components (NfL, MAP2, MBP) are degrading and are not being regenerated by 16 weeks post-implantation. Our more complete data set further questions the validity of using NeuN as the sole marker of neuronal health which has been common practice in the IME literature for some time, as NeuN is not downregulated at the 16WK time point, yet we still see deficits in other neuronal proteins, and 16WKs is associated with chronic recording failure.

#### Autophagy Proteins:

Eight autophagy proteins, including ATG5, BAG3, ULK1, and VPS35, are downregulated in the 8WK and 16WK timepoints compared to naïve control mice (Fig. 1C, D, 5, Table 2). The downregulation of the 8 autophagy proteins indicates that autophagy is not occurring at a healthy rate in implanted mice by 8 weeks post-surgery. Autophagy removes harmful substances from the cytoplasm, allowing for the recovery of injured cells ^26^. Autophagy in neurons is especially important because neurons in the adult motor cortex do not divide or regenerate, so they need to survive the entire lifetime of the organism ^73^. The downregulation of autophagy proteins, along with the fact that neurons around the implant site are still dying up to 16 weeks post-implantation ^56^, suggest that by the 8WK time point the autophagy attempts to save the neurons have failed and neurons are likely resorting to apoptosis or necrosis. Though there are no apoptotic proteins quantified in this experiment, MAP2 is known to undergo proteolysis during apoptosis ^74^. We found MAP2 to be downregulated in both 8WK and 16WK mice compared to naïve control mice (Fig. 1C, D, 4, Table 2). MAP2 downregulation could indicate that apoptosis is occurring in local neurons. Neuronal dieback is a major concern for IME researchers as well as patients, and autophagy could be a target for the prevention of neuronal death. One study found that overexpression of ATG5, an autophagosomal protein found to be downregulated in our experiment, leads to nearly 20% longer lifespans in mice ^75^. A method that promotes autophagy in implanted animals before or during the 8WK and 16WK time points may prevent neuronal dieback over chronic time points.

#### Implications for future studies:

Proteins within the astrocytic, microglial and peripheral immune sections represent pathways that ideally would not be activated following IME implantation. The knockouts of several key inflammatory genes, including CD14 and C3, have been investigated with some success in IME applications. Ki-67, or a different protein involved in proliferation, may be worth exploring at early time points prior to 4 weeks post-implantation. Our previous understanding of immune cell proteomic activity following IME implantation comes largely from histology and western blot studies. Immunohistochemistry has mapped the timelines for microglia, macrophage, and astrocyte aggregation around the implant site. Ravikumar *et al*. found that microglia and macrophage populations peak at acute timepoints (2 weeks post-implantation) and slowly decrease over chronic timepoints (up to 16 weeks post-implantation). Astrocytic aggregation was shown to be highest at acute timepoints (2 weeks post-implantation), then fluctuate slightly around similar values between 4 and 16 weeks post-implantation ^9^. Our analysis confirms microglia and macrophage-related proteins (SPP1, MHC II, IBA1) are upregulated at the 4WK timepoint. By the 8WK time point, SPP1, MHC II, and IBA1 all lose significant differential expression, and F4/80 is a microglial/macrophage protein that becomes significantly upregulated. Astrocytic activation, quantified through GFAP, is consistently upregulated at 4-, 8-, and 16-weeks post-implantation. Other microglia, macrophage, and astrocyte-related proteins such as CD11b-c and CD45 are upregulated at all measured time points of 4-, 8-, and 16-weeks post-implantation. Our analysis confirms that microglia, macrophages, and astrocytes are present at all time points, but brings context into some molecular changes that are occuring in the protein expression of these cells over time.

Our investigation of neuronal health proteins, specifically downregulation of neuronal cytoskeletal and myelin proteins, may be an indication that irreversible damage is being done to neurons surrounding the implant. NeuN, which is typically used as a marker for neuronal health, is restored to healthy levels 50+ mm from the implant site by 16 weeks post implantation ^7^. Our protein expression analysis shows that approximately 62% of NfL is lost in 16WK implanted animals. The 62% of NfL lost within 180 mm is unlikely to be contained within the 50 mm radius where neuronal nuclei are depleted. This calls into question how reliable NeuN is as a marker for neuronal health. It could be that neurons are present around the implant but are not functional. Promoting autophagy, specifically ATG5 or ATG12, could improve neuronal survival or functionality.

Using functional recording studies, we can determine if the timeline of proteomic changes align with the functionality of these devices in vivo. Overall we see in the literature that units recorded and active electrode yield decline over time The observed decline in performance at the 8WK time point does not seem to have a definitive effect on recording at that time point, but may be a turning point for neuronal health that, if prevented, could improve chronic performance beyond 8 weeks post-implantation. Our recommendation for any drug with the intent to prevent neuronal defecits would be to either target continuously up to 8 weeks post-implantation or to targer between the 4WK and 8WK time points, which is when the most damage seems to occur (Fig. 4).

## Methods

### Animal Preparation:

All mice were housed at Case Western Reserve University, and all experimental protocols and procedures were approved by the Case Western Reserve University IACUC committee and performed in compliance with an IACUC-approved protocol. Additionally, all methods are reported in accordance with ARRIVE guidelines. Animals underwent identical surgical procedures following established laboratory protocols ^11,27,107^, resulting in four IME implants spanning both the left and right motor and sensory cortices. During the surgeries, animals were anesthetized with isoflurane. For full surgical details, please see the above references. In brief, four craniotomies were performed, 1.5 mm lateral and 1.0 mm anterior and posterior to the bregma. The probes were inserted 1 mm deep into the cortex, with four implants per animal. Kwik-Sil was placed over the cavity, and dental cement was added to secure the probe.

Implanted mice were sacrificed via cardiac perfusion (described below) at 4, 8, or 16 weeks following their implantation surgery (N=4 for each time point). These groups will be referred to as 4WK, 8WK and 16WK respectively. These mice were compared to naïve control mice (N=4), with no implant or craniotomy.

### Microelectrodes:

The probes in this study were non-functional Michigan-style silicon probes from the Pancrazio and Cogan Laboratories at the University of Texas at Dallas. The electrodes were washed three times in 95% ethanol and sterilized with cold ethylene oxide gas, in accordance with previous protocols ^108,109^. Probes were 2 mm long, 15 μm thick, and 123 μm wide at the widest portion of the shank. The decision to use non-functional probes was due to this study’s focus on the neuroinflammatory response, with plans to explore the link between the protein expression and recording performance in future experiments.

### Perfusion and Tissue Processing:

At predetermined endpoints, mice were anesthetized with ketamine and xylazine. An incision was then made through the diaphragm and ribcage to expose the heart. While the heart was still beating, a butterfly needle attached to a peristaltic pump was inserted into the left ventricle. The pump was turned on, allowing the flow of 1X phosphate-buffered saline (PBS) into the aorta. The right atrium was cut to relieve pressure on the heart and allow the outflow of blood and PBS. Once 30 ml of 1X PBS was pumped, another 30 ml of 30% sucrose solution in PBS was pumped into the animal for cryoprotection of the brain. The brain was extracted, suspended in Optimum Cutting Temperature compound (OCT, Sakura Finetek USA, Item Number: 25608–930), and flash-frozen on dry ice. The brains remained at −80°C until further processing. The brains were then cryo-sectioned into 5 μm sections through the depth of the mouse cortex (~1 mm) and placed on glass microscope slides (Fisher Scientific, Item Number: 12–550-15). Tissue mounted on the slides was stored at −80°C until used for multi-omics experiments; note only proteomics will be reported in this manuscript.

### Analysis for Spatially-Resolved Protein Expression:

Slides were removed from the −80°C freezer and fixed for 16 hours in 10% neutral-buffered formalin. Following fixation, the slides were washed three times, ten minutes each, with 1X Tris-buffered saline with 0.1% Tween 20 detergent (TBST). Next, the slides were transferred to 1X Citrate buffer to undergo antigen retrieval, which consisted of 15 minutes in the TintoRetriever Pressure Cooker (Bio SB, Item Number: BSB 7008) on high temperature and pressure settings. The slides were removed from the pressure cooker and left to sit in the citrate buffer for 25 minutes at room temperature. Next, slides were washed three times in in 1X TBST for ten minutes each. To prepare for overnight staining, a hydrophobic barrier was created on the slide surrounding the tissue using an ImmEdge^®^ Hydrophobic Barrier PAP Pen (Vector Laboratories, Item number: H-4000). Slides were stained with morphology markers for cell types of interest: neuronal nuclear protein (NeuN) for neuronal nuclei and glial fibrillary acidic protein (GFAP) for activated astrocytes. Morphological markers allowed for the selection of regions of interest, which were rings with 180 μm radius from the implant site. Slides were also stained with protein reagents provided by NanoString, which contained antibodies for the proteins quantified in this study attached to a fluorescent sequence unique to each protein. The total staining solution consisted of 1:40 anti-GFAP (Alexa Fluor^®^ 532 GA-5, Item Number: NBP2–33184AF532), 1:100 anti-NeuN (Alexa Fluor^®^ 647 EPR12763, Item Number: ab190565), and 1:25 of all 6 protein panels and modules provided by NanoString (described below). Slides were placed in a humidity chamber for 16 hours in a 4°C refrigerator for incubation.

The proteins quantified in this study were selected based on existing panels sold by NanoString. For every protein in the panel, the reagent provided would include an antibody for that specific protein bound with a UV-cleavable link to a fluorescent “barcode.” This barcode consists of a fluorescent sequence that is unique to the antibody it is bound to. The panels purchased for this study were the Neural Cell Profiling Core (25 proteins, Item Number: 121300120), which was paired with the Glial Cell Subtyping Module (10 proteins, Item Number: 121300125), and Autophagy Module (10 proteins, Item Number: 121300124). This makes for 45 total proteins in the Neural Panel (Table 1). The Immune Cell Profiling Core (23 proteins, Item Number: 121300106) was paired with the Immune Cell Typing Module (7 proteins, Item Number: 121300118) and the Immune Activation Status Module (8 proteins, Item Number: 121300117), for a total of 38 proteins in the Immune Panel. This collection of different cores and modules make up the 83 total proteins quantified in this study. Table 1 lists all the proteins from each panel. CD68, a protein in the Neural Cell Profiling Panel that is used as a marker for activated microglia, was removed from the study because it was unable to be validated after multiple attempts.

Following overnight incubation, slides were washed three times in 1X TBST for ten minutes each. The hydrophobic barrier on the slides was filled with 200 μl of 10% neutral-buffered formalin, and slides were fixed for an additional thirty minutes in the humidity chamber. The slides were washed twice in 1X TBST for five minutes each. Slides were then stained with 1:10 concentration of nuclear stain SYTO-13 (NanoString Technologies, Item Number:121300303) diluted in 1X TBS. Following staining, slides were loaded into the GeoMx Digital Spatial Profiler (NanoString Technologies, Seattle, WA) for region of interest (ROI) selection and protein expression analysis.

Once slides were loaded into the GeoMx Digital Spatial Profiler, the slides were imaged using the fluorescent morphology markers for NeuN and GFAP. These markers allowed us to identify the implant sites and create a 180 μm radius ROI surrounding each implant. The GeoMx Digital Spatial Profiler would then collect the “barcodes” in each ROI one region at a time. This was performed by shining UV light over the entire region of interest, cleaving all the barcodes from the antibodies they were bound to. The cleaved barcodes for each ROI were then collected in one well of a 96-well plate.

The 96-well plates were dried overnight in the GeoMx at room temperature, then were rehydrated in DNAse/RNAse free water. Next, GeoMx Hybridization Codes (NanoString Technologies, Item Number: 121300401) were added to rows A-H to differentiate each row and allow for pooling. Each column was then pooled, creating 12 pooled sample solutions that were loaded into the nCounter MAX/FLEX system (NanoString Technologies, Seattle, WA). The nCounter system transferred the barcodes in the pooled sample solutions into a clear cartridge. The cartridges were then scanned by the nCounter MAX/FLEX system, outputting a count for every barcode found in each specific ROI, which is equivalent to the protein expression in each ROI.

### Statistical analysis:

The raw data with of the protein counts, or bar codes captured from the NanoString nCounter, was exported and analyzed in MATLAB. Differential expression of identified/captured proteins was analyzed using a custom MATLAB script. Each protein-associated fluorescence sequence was normalized with the geometric mean of housekeeping genes. Housekeeping proteins are known to be present with minimal fluctuation in expression between different cells, conditions, and samples ^20^. Normalizing to housekeeping proteins is intended to account for the number of cells and total proteins collected in each run. The normalized count reflects the relative value of proteins expressed in the collected region, allowing us to compare collections with different areas and cell counts against each other. Housekeeping proteins are not included in the differential expression analysis. Negative control probes were used for quality control, as they were antibodies for proteins that are not present in mouse tissue. Housekeeping and negative control counts were not included in the differential expression analysis. Following normalization and quality control, an unpaired t-test was used to compare groups to one another. Unadjusted p-values were corrected using a Benjamini-Hochberg false discovery rate to account for random significance. Neural and Immune Cell Profiling Panels were run separately in MATLAB, except for the eight proteins that were present in both panels: cluster of differentiation 11b (CD11b), cluster of differentiation 31 (CD31), cluster of differentiation 40 (CD40), cluster of differentiation 45 (CD45), cluster of differentiation 163 (CD163), integrin alpha X chain protein/ cluster of differentiation 11c (CD11c), Ki-67, major histocompatibility complex II (MHC II). All eight of the overlapping proteins were immune proteins (Table 1), so the Neural Cell Profiling Panel counts were omitted for these eight proteins, and the counts from the Immune Cell Profiling Panel were analyzed with the rest of the Immune Panel proteins. Data was visualized using volcano plots generated in GraphPad Prism 10 (GraphPad Software, Boston, Massachusetts USA). The volcano plot’s x-axis is the log_2_ of the fold change, which is the average protein count of the implanted group (4WK, 8WK, or 16WK) divided by the average count of the naïve control group. Therefore, all counts are essentially “baselined” to the naïve control group. In this experiment, “upregulated” refers to higher expression of that particular protein in the implanted group with respect to the baseline, or the naïve control group. “Downregulation” would refer to lower expression of that protein compared to baseline. The y-axis is the adjusted p-values (p_adjusted_) from the Benjamini-Hochberg correction, using a significance threshold of p_adjusted_ < 0.05.

## Conclusion

The current study is the first large-scale analysis of proteins surrounding the IME implant site. Previously, several studies have measured mRNA expression following IME implantation and have quantified individual protein expression mainly using fluorescent intensity. This study provides the high-throughput benefits of RNAseq while also quantifying the molecules that are most relevant to the inflammatory process: proteins. The combination of this proteomic expression data with existing transcriptomic studies could allow for both transcription and translation timelines to be mapped for individual genes and proteins. The timeline of protein expression surrounding the implant could be used to create gene therapies to improve chronic IME performance, and our analysis of 4, 8, and 16 weeks post-implantation can provide insight into when therapies would be best administered.

Our dataset is intended to be used as a database for researchers trying to implement specific genetic targets to improve IME performance or reduce brain damage following implantation. Not only can we add new potential targets to the field, such as Ki-67, but researchers looking into specific pathways can reference Fig. 1 and Table 2 to determine the timeline of drug delivery, identify potential dual delivery targets, and get an overview of the other molecules that will be at play during targeting.

One improvement that could be made with this study is the addition of segmentation, either based on distance from the implant or by cell type. Cell segmentation could provide additional context to these results, as many of these proteins are expressed by more than one cell type, and the function of that protein could vary based on the cell type. This would be especially helpful in our analysis of immune cells, which share many common markers (Table 2). Using a finer resolution than 180 mm could also lead to a better understanding surrounding discrepancies in the NeuN marker and other neuron viability markers.

## Figures and Tables

**Table 1:** All proteins included in this study. Columns are split into the Neural and Immune Cell Profiling Panels, which are sets of proteins that were measured and analyzed separately. Negative control proteins are highlighted in orange, and housekeeping proteins are highlighted in blue. All other proteins (highlighted in grey/white) were included in differential expression analysis.

Neural Cell Profiling Panel		Immune Cell Profiling Panel	
Protein Name	Module	Protein Name	Module
**GFAP**	Neural Cell Profiling Core	**GZMB**	Immune Cell Profiling Core
**IBA1**	Neural Cell Profiling Core	**CD11b**	Immune Cell Profiling Core
**Ki-67**	Neural Cell Profiling Core	**CD11c**	Immune Cell Profiling Core
**MAP2**	Neural Cell Profiling Core	**CD19**	Immune Cell Profiling Core
**MHC II**	Neural Cell Profiling Core	**CD3e**	Immune Cell Profiling Core
**MBP**	Neural Cell Profiling Core	**CD4**	Immune Cell Profiling Core
**NeuN**	Neural Cell Profiling Core	**CD45**	Immune Cell Profiling Core
**NfL**	Neural Cell Profiling Core	**CD8a**	Immune Cell Profiling Core
**OLIG2**	Neural Cell Profiling Core	**CTLA4**	Immune Cell Profiling Core
**S100B**	Neural Cell Profiling Core	**F4/80**	Immune Cell Profiling Core
**CD11b**	Neural Cell Profiling Core	**FN**	Immune Cell Profiling Core
**CD163**	Neural Cell Profiling Core	**Ki-67**	Immune Cell Profiling Core
**CD31**	Neural Cell Profiling Core	**MHC II**	Immune Cell Profiling Core
**CD39**	Neural Cell Profiling Core	**PD-1**	Immune Cell Profiling Core
**CD40**	Neural Cell Profiling Core	**PD-L1**	Immune Cell Profiling Core
**CD45**	Neural Cell Profiling Core	**PanCk**	Immune Cell Profiling Core
**CD68**	Neural Cell Profiling Core	**SMA**	Immune Cell Profiling Core
**SYP**	Neural Cell Profiling Core	**CD27**	Immune Activation Status
**TMEM119**	Neural Cell Profiling Core	**CD86**	Immune Activation Status
**ALDH1L1**	Glial Cell Subtyping	**CD127/IL7RA**	Immune Activation Status
**CD9**	Glial Cell Subtyping	**CD40**	Immune Activation Status
**CSF1R**	Glial Cell Subtyping	**CD40L**	Immune Activation Status
**CTSD**	Glial Cell Subtyping	**CD44**	Immune Activation Status
**GPNMB**	Glial Cell Subtyping	**ICOS**	Immune Activation Status
**CD11c**	Glial Cell Subtyping	**BatF3**	Immune Cell Typing
**MSR1**	Glial Cell Subtyping	**CD14**	Immune Cell Typing
**MerTK**	Glial Cell Subtyping	**CD163**	Immune Cell Typing
**SPP1**	Glial Cell Subtyping	**CD28**	Immune Cell Typing
**VIM**	Glial Cell Subtyping	**CD31**	Immune Cell Typing
**ATG12**	Autophagy	**CD34**	Immune Cell Typing
**ATG5**	Autophagy	**FOXP3**	Immune Cell Typing
**BAG3**	Autophagy	**Ly6G/Ly6C**	Immune Cell Typing
**BECN1**	Autophagy		
**LC3B**	Autophagy		
**P62**	Autophagy		
**PLA2G6**	Autophagy		
**TFEB**	Autophagy		
**ULK1**	Autophagy		
**VPS35**	Autophagy		
**Rb IgG**	Neural Cell Profiling Core	**Rb IgG**	Immune Cell Profiling Core
**Rt IgG2a**	Neural Cell Profiling Core	**Rt IgG2a**	Immune Cell Profiling Core
**Rt IgG2b**	Neural Cell Profiling Core	**Rt IgG2b**	Immune Cell Profiling Core
**GAPDH**	Neural Cell Profiling Core	**GAPDH**	Immune Cell Profiling Core
**Histone H3**	Neural Cell Profiling Core	**Histone H3**	Immune Cell Profiling Core
**S6**	Neural Cell Profiling Core	**S6**	Immune Cell Profiling Core

**Table 2:** All proteins included in this study found to show differential expression (DE) in at least one comparison (4WK, 8WK, or 16WK) compared to naïve control. Red/+ indicates upregulation in the implanted group (4WK,8WK,16WK), and blue/− indicates downregulation in the implanted group compared to naïve control mice. White indicates no significant differential expression.

Protein Symbol	Protein Name	Category/Function	DE in 4WK vs. Naïve Control	DE in 8WK vs. Naïve Control	DE in 16WK vs. Naïve Control
**ALDH1L1**	Aldehyde dehydrogenase 1 family member L1	Astrocytes: regulates cell division and growth ^39^			−
**ATG5**	Autophagy related 5	Autophagy: autophagosome protein (KEGG:04140) ^56–58^		−	−
**ATG12**	Autophagy related 12	Autophagy: autophagosome protein (KEGG:04140) ^56–58^		−	
**BAG3**	BAG family molecular chaperone regulator 3	Autophagy: promotes autophagy through increasing glutamine consumption ^76^		−	−
**BECN1**	Beclin-1	Autophagy: forms protein complex that initiates autophagosome formation ^77^		−	−
**CD11b**	Cluster of Differentiation 11b	Microglia, macrophages, peripheral immune: expressed in activated microglia, macrophages, and dendritic cells; plays a role in cell-cell adhesion during inflammation ^40,41^	***+***	***+***	***+***
**CD11c**	Cluster of differentiation 11c	Microglia, macrophages, peripheral immune: integrin protein found in microglia, macrophages, dendritic cells, and T-cells ^41^; CD11c^+^ microglia hypothesized to be neuroprotective ^78,79^;	***+***	***+***	***+***
**CD31**	Cluster of differentiation 31	Endothelial cell marker, mediates BBB permeability, specifically permeability to immune cells ^41^		***+***	
**CD34**	Cluster of differentiation 34	Peripheral immune: Used as a marker for microvascular density ^80^		***+***	
**CD3E**	Cluster of differentiation 3e	Peripheral immune: T-cell receptor ^39^	−		
**CD40L**	Cluster of differentiation 40 ligand	Peripheral immune: present on surface of T-cells, B-cells, platelets and is involved in microglial activation ^81,82^		***+***	
**CD44**	Cluster of differentiation 44	Astrocytes, microglia, macrophages, peripheral immune: marker for activated T-cells, B-cells, and mononuclear cells ^83,84^		***+***	
**CD45**	Cluster of differentiation 45	Microglia, macrophages, peripheral immune: Expressed highly in macrophages, less so in microglia, involved in adhesion and T-cell activation ^85^	***+***	***+***	***+***
**CD86**	Cluster of differentiation 86	Microglia, macrophages, peripheral immune: Present on antigen presenting cells (such as microglia and macrophages), activates T-cells ^86^	−		
**CTLA4**	Cytotoxic T-lymphocyte associated protein 4	Peripheral immune: Prevents or inhibits T-cell activation ^87,88^	−		
**CTSD**	Cathepsin D	Astrocytes, microglia, macrophage, peripheral immune: Regulates proteolysis in lysosomes: expressed in neurons, astrocytes, microglia, and macrophages ^88–91^		***+***	
**F4/80**	Cell surface glycoprotein F4/80	Microglia, macrophages, peripheral immune: Involved in microglial and macrophage activation in mice, but not confirmed in human tissue ^92^		***+***	
**FN**	Fibronectin	Peripheral Immune: Serum protein, likely indicative of BBB permeability, takes on a neuroprotective role, inhibiting apoptosis after brain injury ^93^	***+***	***+***	
**GFAP**	Glial fibrillary acidic protein	Astrocytes: Intermediate filament protein that serves as a marker for activated astrocytes ^94^	***+***	***+***	***+***
**IBA1**	Ionized calcium binding adapter molecule 1	Microglia, macrophages, peripheral immune: Involved in phagocytosis ^40,45^	***+***		
**Ki-67**	N/A	Astrocytes, microglia, macrophages, peripheral immune: Marker of cell proliferation ^41,52^	***+***		
**Ly6G/Ly6C**	Lymphocyte antigen 6 complex, locus G, lymphocyte antigen 6 complex, locus C1	Peripheral immune: Surface protein on neutrophils ^95^	−		
**MAP2**	Microtubule associated protein 2	Neuronal health: Cytoskeletal/microtubule structure ^96^		−	−
**MerTK**	Tyrosine-protein kinase Mer	Astrocytes, microglia, macrophages, peripheral immune: anti-inflammatory, involved in phagocytosis of apoptotic cells; also plays a role in maintaining BBB integrity ^72,97,98^			−
**MHC II**	Major histocompatibility complex Class II	Astrocytes, microglia, macrophages, peripheral immune: Marker of antigen presenting cell, including microglia, astrocytes, and macrophages ^48,49^.	**+**		
**MSR1**	Macrophage scavenger receptor 1	Astrocytes, microglia, macrophages, peripheral immune: proinflammatory, essential for phagocytosis ^99,100^			−
**MBP**	Myelin basic protein	Neuronal health: Essential for proper myelin membrane formation ^69^	−	−	−
**NeuN**	Neuronal nuclear protein	Neuronal health: Binds to DNA in mature neurons, serves as a marker for neuronal nuclei ^65^		−	
**NfL**	Neurofilament light	Neuronal health: Cytoskeletal protein found in neurons ^101^		−	−
**OLIG2**	Oligodendrocyte transcription factor 2	Neuronal health: Promotes myelin production in oligodendrocytes as well as oligodendrocyte differentiation ^102^		−	−
**PanCk**	Pan cytokeratin	Peripheral immune: Marker for epithelial cells ^103^	**+**		
**P62**	Seguestosome-1	Autophagy: Tags protein inclusion bodies and delivers them to autophagosomes for degradation ^104^		−	−
**PLA2G6**	Phospholipase A2 group VI	Autophagy: Essential for metabolism of phospholipids, proper mitochondrial function, inhibition of apoptosis ^105^		−	−
**SPP1**	Secreted Phosphoprotein 1	Astrocytes, microglia, macrophages, peripheral immune: Pro-inflammatory signaling protein expressed by astrocytes, microglia, macrophages, and T-cells ^50^	**+**		
**SYP**	Synaptophysin	Neuronal health: synaptic vesicle protein ^25^		−	
**TFEB**	Transcription factor EB	Autophagy: promotes transcription of genes that lead to autophagosome formation ^106^		−	−
**TMEM119**	Transmembrane protein 119	Microglia: Marker for microglia, specifically resting; function unknown ^40^	**+**		
**ULK1**	Unc-51-like kinase 1	Autophagy: forms protein complex that, initiates autophagosome formation ^77^		−	−
**VIM**	Vimentin	Astrocytes: Intermediate filament protein that regulates astrocyte structure during gliosis, also expressed in endothelial cells ^35,37,38^	−	−	−
**VPS35**	Vacuolar protein sorting 35	Autophagy: forms protein complex that initiates autophagosome formation ^77^		−	−
